# Ammonium Polyphosphate Promotes Maize Growth and Phosphorus Uptake by Altering Root Properties

**DOI:** 10.3390/plants13233407

**Published:** 2024-12-04

**Authors:** Siqi Dong, Asante-Badu Bismark, Songsong Li, Qiang Gao, Xue Zhou, Cuilan Li

**Affiliations:** Key Laboratory of Sustainable Utilization of Soil Resources in The Commodity Grain Bases of Jilin Province, College of Resource and Environmental Sciences, Jilin Agricultural University, Changchun 130118, China; dongsiqi@mails.jlau.edu.cn (S.D.);

**Keywords:** phosphate fertilizer, maize yield, P use efficiency, root morphology

## Abstract

Phosphorus (P) is an essential nutrient for maize growth, significantly affecting both yield and quality. Despite the typically high concentration of available P in black soils, the efficiency of crop uptake and utilization remains relatively low. This study aimed to evaluate the effects of different P fertilizers on maize yield, root growth parameters, and P use efficiency to identify strategies for optimizing P management in black soil regions. Field experiment results indicated that the combination of ammonium polyphosphate (APP) with other P fertilizers led to variations in yield and P fertilizer absorption efficiency. Various P fertilizers were tested, including diammonium phosphate (DAP), ammonium polyphosphate (APP), fused calcium magnesium phosphate (FCMP), a combination of DAP and FCMP (DAP+FCMP), and a control with no phosphate (CK). The results indicated that P application significantly increased maize yield, with APP (171.8 g/plant) outperforming other P application treatments. Different P fertilizer types significantly affect soil P content and the composition of P fractions. APP significantly increased both the total P (TP) and the proportion of inorganic P (Pi). Furthermore, APP application significantly improved root length (RL), surface area (SAR), and root activity (RA) compared to CK, leading to enhanced nutrient absorption. APP also significantly increased P uptake and utilization (REp, FPp, AEp, PHI, and PAC). In summary, by optimizing plant biomass and P uptake, APP can directly and indirectly influence maize yield. Improving rhizosphere properties through the selection of suitable fertilizer types can enhance fertilizer use efficiency and increase maize production.

## 1. Introduction

The global population is projected to grow to 9.7 billion by 2050, with food demand expected to increase by approximately 50% compared to 2012 [1,2]. Against the backdrop of global climate change, the growth and productivity of major staple crops such as rice, wheat, maize, and soybeans face severe threats. The accelerating pace of global climate change is expected to further reduce the productivity of the world’s major food crops. Maize (*Zea mays* L.) is one of the most important crops for food, feed, and energy worldwide. It is not only crucial for food security but also plays a significant role in national socio-economic development [3]. As the most important cereal crop, China’s maize planting area has rapidly increased from 23.056 million hectares in 2000 to 44.2265 million hectares in 2024. Maize is the main driver of grain production growth, and increasing maize yield is a key strategy to ensure China’s food security and alleviate supply–demand imbalances. Given the limitation on the expansion of arable land, efficient fertilization to enhance per-unit yield has become the primary solution to meet food demand [4].

Phosphorus (P) plays a crucial role in agriculture, being indispensable for plant growth and development [5]. It is fundamental to ATP synthesis, photosynthesis, energy transfer, and nutrient transport. Additionally, P is a key component of nucleic acids and phospholipids, which are essential for the structure and function of plant cells [6,7]. Adequate P levels are required for proper root development, flowering, seed formation, and overall plant vigor [8]. Soil available P is primarily distributed in the topsoil and has low availability, with approximately 67% of the world’s land area affected by P deficiency [9]. Moreover, P is one of the key limiting nutrients for crop production, with P deficiency constraining 30% of global crop yields [10]. In China, P deficiency affects 35–40% of maize yields. China has proposed strategies for reducing fertilizer use while increasing efficiency and promoting green agriculture. However, P fertilizer application in China remains high, reaching 16.15 million tons in 2023 [11]. However, in Northeast China, only approximately 20% of the applied P fertilizer is utilized during the growing season, leading to a significant P surplus in the soil [12,13]. Although the high organic matter (OM) content in black soil enhances nutrient availability, it can also lead to the formation of complex compounds with P, thereby reducing its direct availability to plants. Additionally, highly active microbial processes can immobilize P, making it less accessible for plant uptake [14,15,16]. Excessive amounts of other nutrients can interfere with P absorption. The large input of fertilizers and improper fertilization practices have significantly reduced both the yield-enhancing effects and the utilization efficiency of fertilizers, leading to diminishing returns. The utilization efficiency of P fertilizer is only 10–25%, far below the levels seen in developed countries [17,18]. Therefore, it is imperative to innovate and develop new methods to fundamentally change unsustainable practices in agricultural production. This includes reducing nutrient inputs, improving nutrient use efficiency, and lowering potential environmental pollution risks to address challenges related to food security, economic growth, ecosystem sustainability, and social stability.

Previous studies have indicated that various types of P fertilizers, such as DAP, APP, calcium superphosphate (CSP), and calcium magnesium phosphate (CMP), exhibit different effects depending on soil and crop conditions [19]. Specifically, APP is characterized by its high solubility, making it particularly suitable for liquid fertilizers with elevated P content. It also possesses a chelating effect on metal ions, which inhibits the formation of precipitates with P. Upon application to soil, APP undergoes slow hydrolysis into orthophosphate, which is then absorbed and utilized by plants. Consequently, ammonium polyphosphate (APP) has a notably high P utilization rate, classifying it as a long-acting fertilizer [20,21]. Diammonium phosphate (DAP) is effective in enhancing early plant vigor and promoting robust growth, while fused calcium magnesium phosphate (FCMP), as a slow-release fertilizer, provides a stable P supply and reduces the risks of leaching and fixation [14,15,22,23]. Root systems are crucial for soil exploration and nutrient acquisition in sustainable crop production. An optimal root architecture and function contribute to enhanced crop yields and nutrient use efficiency [24]. Root architecture determines the spatiotemporal distribution of roots. Optimal spatial distribution of roots can increase the contact area between roots and soil, expanding the range of soil water and nutrient absorption. It also exhibits high plasticity in response to the distribution of soil water and nutrients [4]. Many studies have looked at the changes in soil AP following the application of P fertilizer [25]. Furthermore, numerous studies have also been conducted on the dynamics of P transformation in soil systems, as well as its fixation and release characteristics, but in practice, the most important question is how much P can be made accessible to crop production from the native soil pool. Since the fertilizer reaction product is the source of P from the soil, understanding how the applied P transforms into specific organic and inorganic forms is important [26]. In addition, several studies have investigated the dynamics of plant nutrients in a variety of soils, but none have investigated the dynamics of P transformation in black soils in Northeast China. Therefore, improving crop root architecture and function to promote the spatial coupling of roots with soil water and nutrients can enhance the efficient utilization of soil water and nutrients by plants [27,28]. However, there is still a lack of systematic research on the specific effects of these P fertilizers in black soil, particularly regarding their comprehensive impact on maize growth and P utilization efficiency.

In modern agricultural production, traditional diammonium phosphate (DAP) has high solubility, making it prone to loss, fixation in the soil, and poor mobility, which severely limits the availability of P to the crop rhizosphere. This is one of the major reasons for the low P fertilizer use efficiency in China. Extensive research has investigated the impact of P application on maize yield [29,30].

However, the specific changes in P fertilizer types needed to improve utilization efficiency and establish effective mechanisms of P use remain unclear. Our previous field experiments have demonstrated that different proportions of P fertilizers significantly increased maize yield and P utilization efficiency, with the most significant effects observed in the APP treatment. Based on this result (Supplementary Information), pot experiments were conducted to further elucidate the effects and driving factors of different P fertilizers on maize yield and P fertilizer utilization rate. This study employed various sources of P fertilizer (DAP, APP, and FCMP) to investigate their impact on maize growth and yield parameters over a 120-day growth period. Measurements of crop yield and plant samples were conducted, along with analyses of soil P forms, to examine the relationship between crop growth, yield, and soil P characteristics. Specifically, this study aimed to investigate the physiological and rhizosphere responses of maize to these P sources and to further elucidate the factors driving yield improvement, providing insights into strategies for improving P fertilizer utilization efficiency in black soil regions.

## 2. Results

### 2.1. The Impact of Different P Application Strategies on Maize Yield

**Figure 1:**
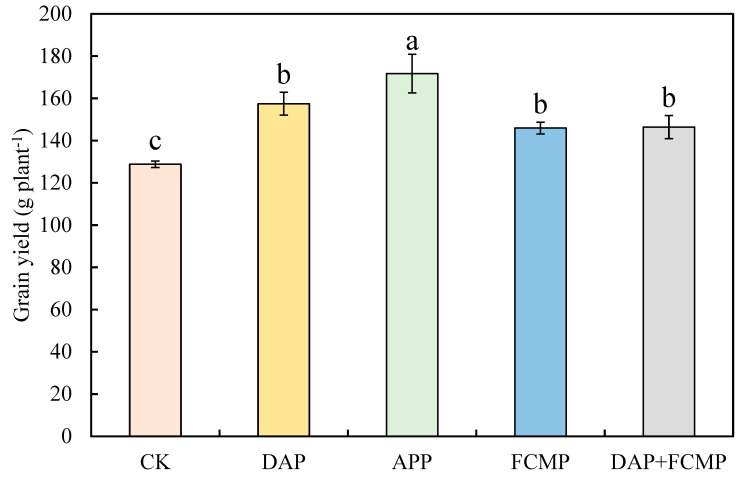
Maize yield as affected by P treatments. Effect of P on maize yield. DAP (diammonium phosphate), APP (ammonium polyphosphate), FCMP (fused calcium magnesium phosphate), DAP+FCMP (diammonium phosphate combined with fused calcium magnesium phosphate), and CK (no phosphate). The data are the means of three replicates, and the error bars represent the standard deviations. Different letters indicate significant differences (*p* < 0.05).

The application of P fertilizer had a significant positive effect on maize yield, as shown in Figure 1. Specifically, the yield of maize plants under P supply fertilizer consistently exceeded that of the control treatment (CK), which had a yield of 128.8 g plant^−1^. The maize plants treated with P exhibited an increase in yield ranging from 3.03% to 21.77%. The highest yield was observed under the APP, where the plants produced 171.8 g plant^−1^. This increase in yield highlights the importance of P in enhancing maize growth, with the APP treatment showing the most pronounced positive impact.

### 2.2. The Impact of Different P Application Strategies on the Forms of P in Soil

**Figure 2:**
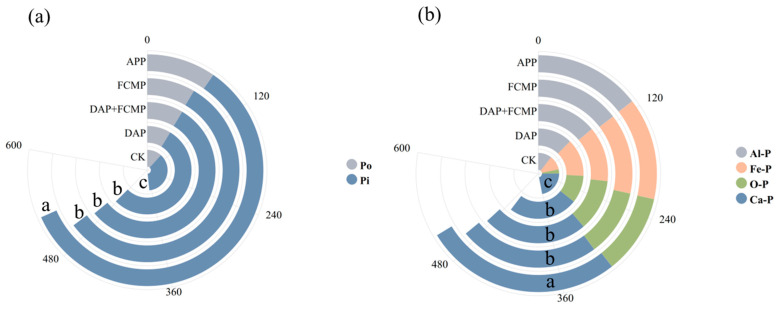
The influence of P fertilizer types on the fractionation and transformation of TP (**a**) and Pi (**b**) in black soil. DAP (diammonium phosphate), APP (ammonium polyphosphate), FCMP (fused calcium magnesium phosphate), DAP+FCMP (diammonium phosphate combined with fused calcium magnesium phosphate), and CK (no phosphate). Different letters indicate significant differences (*p* < 0.05).

Figure 2a illustrates the impact of various P supplies on the composition of Po and Pi in TP. The TP varies widely under different P supply, with the APP treatment showing a significantly higher TP than other treatments by 5.5–41.4% (*p* < 0.05). The proportion of Pi was higher than that of Po in all treatments, making up 65.0–78.9% of the TP, with P in the APP and FCMP treatments exceeding that of other treatments and the CK treatment having the lowest Pi content. Figure 2b presents the classified composition Pi. Ca-P was predominant in all treatments, comprising 41.0% of the Pi, particularly in the APP treatment, where the Ca-P content was significantly higher than in other treatments. The O-P ratio was the smallest, accounting for only 15.8% of the Pi. Statistical analysis reveals significant differences in the composition and classification of soil P among the various treatments.

### 2.3. The Impact of Different P Application Strategies on Dry Matter Biomass Accumulation

**Figure 3:**
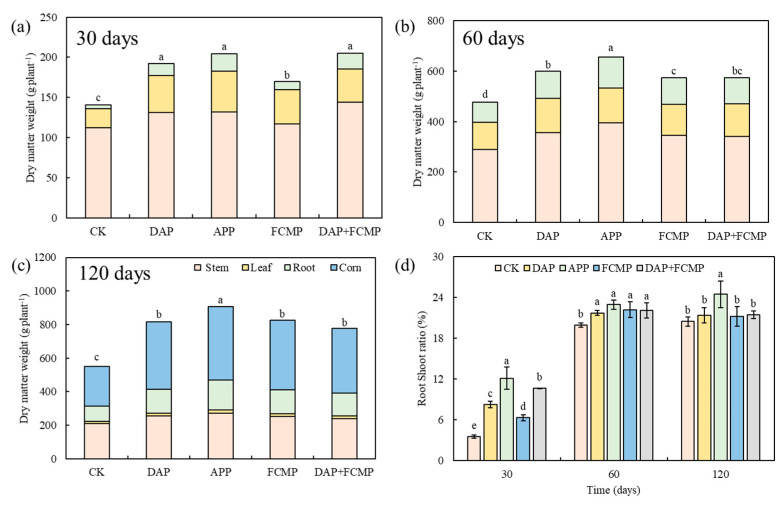
Effects of P fertilizer types on maize biomass at 30 (**a**), 60 (**b**), and 120 (**c**) days and root shoot ratio (**d**). DAP (diammonium phosphate), APP (ammonium polyphosphate), FCMP (fused calcium magnesium phosphate), DAP+FCMP (diammonium phosphate combined with fused calcium magnesium phosphate), and CK (no phosphate). Vertical bars denote the standard deviation of the mean. Different letters indicate significant differences (*p* < 0.05).

The dry matter biomass of the stem was consistently higher than that of the leaves at 30-, 60-, and 120-day intervals. At maturity (120 days), grain weight was the highest, followed by stem and root weights. Under different P treatments, the dry matter biomass accumulation and total accumulation of various organs in corn remained consistent, with the highest levels observed in the APP treatment (Figure 3). The dry matter biomass of each organ was significantly higher in the APP treatment throughout the growth period compared to other treatments, showing an increase of 9.8–65.0%. At 60 days, the root-to-shoot ratio in the P treatment was significantly higher than in the CK. At 30 and 120 days, the root-to-shoot ratio in the APP treatment was significantly higher than in the other treatments (Figure 3).

### 2.4. The Impact of Different P Application Strategies on P Uptake

**Figure 4:**
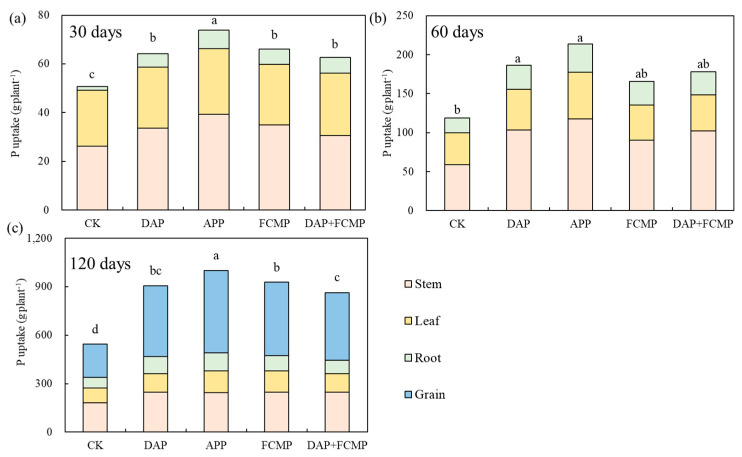
Effects of P fertilizer types on the P uptake in different maize organs at 30 (**a**), 60 (**b**), and 120 (**c**) days. DAP (diammonium phosphate), APP (ammonium polyphosphate), FCMP (fused calcium magnesium phosphate), DAP+FCMP (diammonium phosphate combined with fused calcium magnesium phosphate), and CK (no phosphate). Vertical bars denote the standard deviation of the mean. Different letters indicate significant differences (*p* < 0.05).

During the 30- and 60-day growth periods, P absorption in maize stems exceeded that in leaves and roots. However, at 120 days, P accumulation in maize grains surpassed that in leaves and roots. Significant differences were also observed among treatments involving different types of P fertilizers (Figure 4). Throughout the entire growth period, P absorption was highest with the application of APP (1000.83 g plant^−1^), followed by FCMP (928.23 g plant^−1^) and DAP (906.51 g plant^−1^) (Figure 4).

### 2.5. The Impact of Different P Application Strategies on P Use Efficiency

Fertilizer use efficiency was a critical indicator for evaluating the rational application of fertilizers. It described the utilization of P fertilizers by crops from various perspectives. The effects of different treatments on P fertilizer use efficiency are presented in Table 1. Observations from Table 1 reveal significant disparities in Rep (P fertilizer utilization efficiency), FPp (P partial factor productivity), AEp (P partial factor productivity), and PHI (P harvest index) across the treatments.

Specifically, the APP treatment exhibited a significantly elevated REp of 35.12% compared to the other P treatments, whereas the DAP+FCMP treatment manifested the lowest REp at 24.37%. In terms of FPp, the APP treatment demonstrated a markedly higher value at 132.13 g/g, with no significant differences observed among the other P treatments (DAP, FCMP, and DAP+FCMP), among which FCMP recorded the lowest FPp at 112.27 g/g. The pattern of AEp mirrored that of FPp. Furthermore, the PHI was notably higher in the APP treatment at 51.01%, compared to the other P treatments. In summary, the application of APP as a foundational fertilizer enhanced REp, FPp, AEp, and PHI. The soil P activation coefficient (PAC) was the ratio of AP to TP, reflecting the activity level of soil P. In Table 1, the PAC for the CK treatment was 5.65, which was significantly reduced by 25.3% compared to the P application. The PAC of the APP treatment was significantly higher than that of the other P fertilizer treatments by 16.10%. These results suggest that the APP treatment was beneficial in enhancing the PAC.

### 2.6. The Impact of Different P Application Strategies on Maize Root Activity

**Figure 5:**
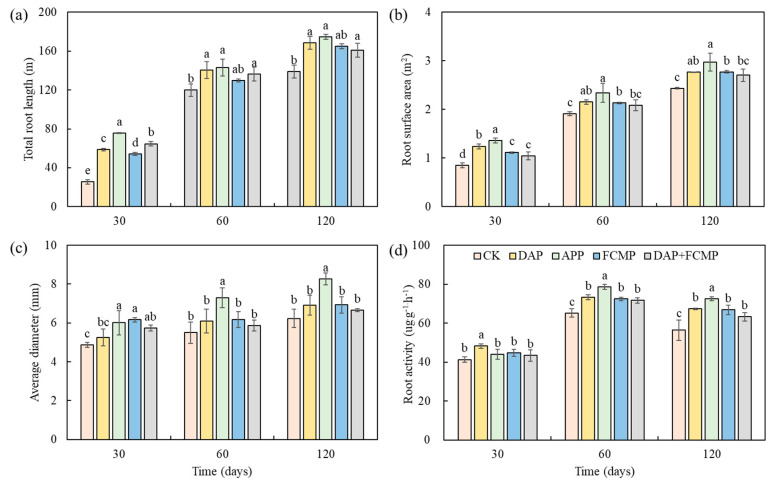
Effects of P fertilizer types on total root length (**a**), root surface area (**b**), root diameter (**c**) and root activity (**d**) of maize at 30, 60, and 120 days. DAP (diammonium phosphate), APP (ammonium polyphosphate), FCMP (fused calcium magnesium phosphate), DAP+FCMP (diammonium phosphate combined with fused calcium magnesium phosphate), and CK (no phosphate). Vertical bars denote the standard deviation of the mean. Different letters indicate significant differences (*p* < 0.05).

Figure 4 illustrates the impact of P application on maize root system morphology. At the 30-day mark, RL in the P fertilizer treatments was significantly greater than in the CK treatment. Specifically, total root lengh (RL) in the APP treatment was notably higher than in the other P fertilizer treatments. Although RL in the APP treatment remained higher at the 60- and 120-day marks, the differences were not statistically significant. This indicates that P application can enhance maize root morphology, which is beneficial for growth. Similar results were observed for root surface area (SAR) and root diameter (RD) (Figure 5).

RA at the 120-day was notably higher than at the 30- and 60-day (Figure 5). At the 30-day mark, RA in the DAP treatment was significantly greater than in all other treatments. Furthermore, RA in the APP treatment was markedly the highest at both 60- and 120-day intervals.

### 2.7. The Mechanism of P Fertilizer Driving Maize Growth Characteristics on Maize Yield

**Figure 6:**
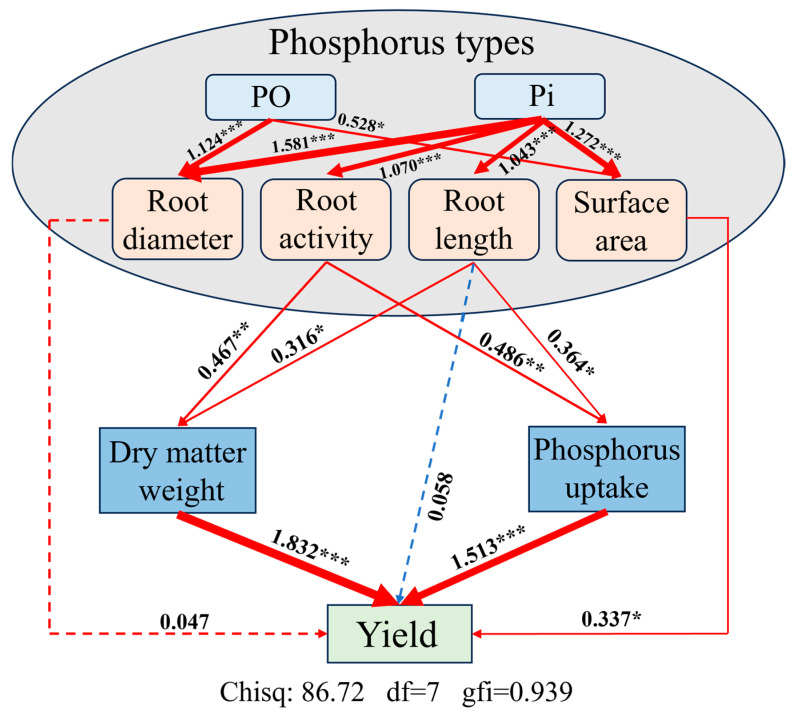
Structural Equation Modeling (SEM) between yield, crop P indicators, and root morphology dates. P types are represented as APP and other P applications. The red line represents positive correlation, and the blue line represents negative correlation. * indicates significance between root morphology or crops P index and maize yield (*, *p* < 0.05; **, *p* < 0.01; and ***, *p* < 0.001).

SEM was employed to analyze the direct and indirect effects of soil P forms, root growth indicators, plant dry matter biomass, and P uptake on maize yields. The SEM analysis produced a chi-square (χ^2^) value of 117.930, df of 17, and a goodness-of-fit index (GFI) of 0.939 for maize yields (Figure 6). SEM analysis revealed that P application significantly impacted soil P, including both Po and Pi. The regulation of Pi on RA and RL directly and indirectly affects maize yield through plant dry matter weight and P uptake (Figure 6). Among these indicators, SAR was recognized as the most critical factor, directly affecting maize yield due to its individual effects (path coefficient = 0.337). Both RL and RA influence maize yield by regulating plant dry matter weight and P uptake. The positive impact of plant dry matter weight on maize yield was the largest (path coefficient = 1.832), followed by P uptake (path coefficient = 1.513) (Figure 6). In addition, compared to other factors, RA has a significantly higher indirect impact on maize yield. For instance, RA directly impacts dry matter weight (path coefficient = 0.467) and P uptake (path coefficient = 0.486), thereby ultimately regulating maize yield (Figure 6).

## 3. Discussion

As an important nutrient element in soil, P plays a crucial role in plant reproductive growth and the development of flowers and fruits. The P content and its form in the soil directly affect soil P supply capacity and fertility. Long-term fertilizer application and continuous tillage may lead to a decrease in soil fertility, resulting in a significant increase in TP content while reducing AP [31]. Therefore, enhancing the soil’s ability to activate P and reducing excessive dependence on fertilizers for production is key to maintaining sustainable soil production. The form in which P exists in the soil directly affects its availability. P in the soil is mainly divided into two categories: Pi and PO. In agricultural soils, Pi accounts for about 60% to 80% of TP [32,33,34]. Pi components include mineral-bound P, adsorbed Pi, and Pi in soil solution. The majority of Pi absorbed and utilized by crops comes from Pi in the soil solution [35,36]. The application of new fertilizers generally increases the AP in the soil, which has a positive impact on crop root growth, especially for maize. Especially APP and DAP, which have a slow release rate, typically exist in the form of water-soluble and exchangeable Pi. The main advantage is their ability to dissolve phosphates formed with calcium, magnesium, and other ions in the soil, enhancing P availability and allowing for rapid absorption by plants, especially for maize, which has a high P demand [19]. Our study also found similar results, where P fertilizer application increased the TP content in the soil, especially with the APP treatment, which increased the proportion of Pi in TP and significantly improved the P activation coefficient (Figure 2, Table 1). A possible reason is that organic Po content usually changes little, as the release process of Po is slow. APP does not directly affect the transformation of Po, but it may indirectly promote the conversion of Po by increasing the soil pH. Due to its higher water-soluble P content, APP can provide absorbable P sources more quickly, helping to increase the level of AP in the soil, thus promoting the growth of crops such as maize. For farm managers, it is essential to choose the right type of P fertilizer and apply it based on the soil’s P supply status, as this is key to improving crop P absorption efficiency and promoting root growth [37].

Our findings reveal that maize root systems exhibit varied responses to different types of P fertilizers, which are associated with enhanced nutrient uptake and promoted growth. The application of different P fertilizers significantly altered the morphological indices of maize roots. The root-to-shoot ratio is commonly used to assess plant growth, reflecting the correlation between the belowground and aboveground parts of the plant. P facilitates root growth and development and, when adequately supplied, can increase the root-to-shoot ratio [38]. Our study found that at the time of maize harvest, the root-to-shoot ratio of P-treated maize increased by 3.7–19.6% compared to untreated maize (Figure 3). Research corroborates our findings, showing a significant increase in the root-to-shoot ratio for all P treatments compared to the control, indicating that P promotes biomass allocation to the roots [39]. The root-to-shoot ratio of APP was the highest (24.47%) among all P application treatments in the later growth stage (120 days), consistent with the results described by Luis [40], which showed that the root-to-shoot ratio of maize treated with APP was significantly higher than that of maize using conventional P fertilizers in silty loam and sandy soils, indicating a more pronounced root growth relative to stem growth.

Balancing the growth of stems and roots may serve as a strategy to enhance P uptake in crops [41]. Previous research indicates that P plays a crucial role in the growth of both stems and roots in crops [42]. Our findings demonstrate that in P-fertilized treatments, the RL, SAR, RD, and RA of maize were significantly higher compared to treatments without P application (Figure 5). Consistent with previous studies, P application has been shown to increase root weight and length in crops grown in Brazilian Oxisol soils [43]. Furthermore, other studies have confirmed that as maize absorbs P, there is an improvement in root distribution quality and morphology, as well as in root volume and the root-to-canopy surface area ratio [44]. Wang et al. found that the properties of maize roots are significantly influenced by the type of P fertilizer applied [45]. The application of APP increased the density and length of lateral roots, significantly enhancing P uptake efficiency. This effect may be attributed to the varying N/P ratios among different P fertilizers. Root proliferation enhances the contact area between roots and soil, thereby increasing nutrient absorption. Nutrient supply can significantly promote root growth, including increased RL and RD [46,47]. Enhancing root–soil interactions and improving the ability of roots to acquire soil nutrients is crucial for sustainable production [48].

Our work findings indicate that the application of APP enhances P absorption in maize, thereby increasing plant dry matter biomass and promoting root dry matter biomass allocation (Figure 2 and Figure 3). Due to differences in solubility, plant availability, and soil interactions, different forms of P fertilizers have varying effects on maize aboveground biomass. Studies have shown that water-soluble P fertilizers, such as APP, monoammonium phosphate (MAP), and urea phosphate (UP), facilitate the accumulation of aboveground biomass and the translocation of dry matter biomass within plants [20]. This is corroborated by recent research, which found that the application of polyphosphates (PolyPs) significantly increased P uptake in both the aboveground parts and roots of maize [45]. Both APP and PolyPs are compounds where P is the main component, primarily used in agriculture as fertilizers to provide the essential P needed by plants. They contain multiple phosphate units connected by covalent bonds to form long chains or cyclic structures. This polyphosphate structure allows for the slow release of P, enhancing plant P uptake efficiency [37,40].

We found that, compared to control plants without P, the application of APP increased P uptake by 83.9% and improved P uptake by 7.8–16.2% compared to other P treatments (Figure 4), significantly increasing plant dry matter biomass (Figure 3). This aligns with the results obtained by Gao et al. [37], where maize plants fertilized with polyphosphates demonstrated enhanced dry matter accumulation. Additionally, a study found a significant correlation between total dry matter biomass, P uptake, and root characteristics of maize plants under polyphosphate supply. This concurs with our results (Table S2), where the application of APP enhanced plant dry matter biomass. Specifically, APP increased dry matter biomass by 65.0% compared to unfertilized plants and by 9.8–16.7% compared to other P treatments (Figure 3). APP aids in chelating micronutrients, reducing P fixation in the soil and promoting root development. Consequently, it facilitates maize growth, enhancing P nutrient uptake and utilization to achieve higher dry matter biomass [48,49].

The differences in maize yield among the different P fertilizer treatments were significant (Figure 1), which aligns with the findings of Mohammad et al. [50]. However, some authors have reported that P fertilizer application does not significantly affect grain yield [51,52]. The discrepancy could potentially be attributed to the ample P availability in the soil prior to the experiment, which was adequate to satisfy the growth demands of the crop [53,54]. Our results regarding maize yield are consistent with those of Aulakh et al. [55], Bai [56], and Ahmed [57], who explained that greater P and nitrogen contributions provided by the APP treatment resulted in larger and more robust grains.

Our findings indicate that the application of different P fertilizers can effectively modulate crop root system characteristics, thereby improving the efficient absorption and utilization of soil nutrients, leading to higher crop yields. P fertilizers promote root growth by increasing RL, RD, and SAR, thereby enhancing the plant’s ability to acquire nutrients from the soil (Figure 6). Research indicated that P applications can significantly enhance P content in maize tissues, which is crucial for various physiological functions. Sufficient P nutrition results in increased dry matter accumulation and overall plant biomass. Amin [58] demonstrated that the use of APP significantly increased maize dry matter biomass through improvements in root characteristics, directly linked to higher biomass yield. P fertilizers also improve plant tolerance to abiotic stresses, such as drought and cold, by enhancing root development and overall plant vigor. Wang [59] showed that P fertilizer application can boost maize resistance to environmental stresses, helping achieve more stable yields across diverse conditions.

In conclusion, while improving P use efficiency through the application of different P fertilizers to increase yields, it is essential to consider the compatibility between crops, soil, and fertilizers. Selecting the appropriate type of fertilizer, along with its application rate and method, can enhance fertilizer use efficiency. The type of P fertilizer had a significant impact on maize growth and biomass. Suitable P fertilizers can stimulate plant growth and increase yield. The correlation between P acquisition efficiency and root parameters explains why the application of novel P fertilizers, particularly APP, promotes maize growth.

## 4. Materials and Methods

### 4.1. Experimental Design

This research was conducted in the greenhouse facility of Jilin Agricultural University, located in Changchun, Jilin Province, Northeastern China. The region experiences an average annual rainfall of approximately 600–700 mm, with 55% of this precipitation occurring between July and September. The climate was characterized as a continental monsoon with subhumid conditions. The average annual temperature stands at 8 °C. The test soil was collected from the experimental field on the campus of Jilin Agricultural University (43°48′36″ N, 125°24′51″ E). This soil is classified as typical black soil [60]. Before the experiment, the physical and chemical properties of the soil were as follows: OM 25.02 g/kg, pH 5.85, TP 0.36 g/kg, AP 12.2 mg/kg, and available potassium (AK) 190.0 mg/kg.

The pot experiment was conducted from June 2020 to October 2020. The experiment comprised five treatments, with 12 replicates per treatment, resulting in a total of 60 pots. P fertilizers were prepared in the laboratory by separating them into nutrient solutions and adding them in the appropriate order. Calcium magnesium P, superphosphate, and other incompletely dissolved P fertilizers were ground, sifted for 2 mm, and evenly mixed. The different P fertilizers were applied to 13 kg of black soil per pot at the following rates: (1) diammonium phosphate (DAP, 100 mg kg^−1^), (2) ammonium polyphosphate (APP, 100 mg P kg^−1^), (3) fused calcium magnesium phosphate (FCMP, 100 mg P kg^−1^), (4) DAP combined with FCMP (DAP+FCMP, 1:1 ratio), and (5) no phosphate (CK). In all the treatments, urea was used to adjust the nitrogen level to a consistent rate of 200 mg N kg^−1^. The maize variety tested was Zhengdan 958. Seeds of uniform size were selected and disinfected by soaking in a 10% H_2_O_2_ solution for 30 min, followed by thorough rinsing with deionized water. After 7 days of emergence, two seedlings with consistent growth were retained. During the experiment, soil moisture content was maintained at 70% of field capacity by weighing every 2 to 3 days to ensure adequate water supply, irrespective of differences in plant dry weight.

### 4.2. Sample Collection and Test

The yield assessment was conducted during the maize harvesting phase using the following methodology. The number of maize grains per ear was determined by counting the grains in both rows and columns. Subsequently, the grains were weighed after threshing to determine the yield.

The plants were harvested at 30-, 60-, and 120-day intervals after planting by cutting them at the stem base with scissors and carefully removing the complete root systems. The harvested parts were separated into stems, leaves, roots, and grains (at 120 days). These samples were then initially treated by killing at 105 °C for 30 min, followed by drying to a constant weight at 75 °C in a drying oven to accurately determine the TP concentration and dry weight. Maize root samples were also collected simultaneously at 30, 60, and 120 days. The length and width of the roots were measured using a ruler or measuring tape. RA was assessed using the methylene blue solution, and the P concentration in the solution was determined using a spectrophotometer at a wavelength of 660 nm. Root image analyses were performed with the EPSON GT-X970 scanner (Epson GT-X970, Seiko Epson Corp., Nagano, Japan). Root images were analyzed for RL, SAR, and RD by using WinRHIZO software (V5.0, 2016 a Regent Instruments, Quebec, QC, Canada).

Soil samples were taken at 0, 30, 60, and 120 days after sowing. The soil sampling was initially sieved with a 2 mm sieve and separated from roots, and then the soil was fully mixed. After air drying, the soil was screened according to the requirements of different measurement indexes. The AP was determined using 50 mL of NaHCO_3_ solution following the AP extraction method [61]. Soil TP was obtained using the block digest procedure. OP in the soil was established using the ignition method. OP concentration was determined by the difference between the P content in the ignited sample and the P content in the unignited sample. The determination of Pi fractions in the soil was carried out using the methods outlined for the classification of neutral soils as adopted in the present experiment. The procedure followed the extraction of the soil with 1 N NH_4_Cl, 0.5 N NH4F, 0.1 N NaOH, 0.5 N H_2_SO_4_, 0.3 N sodium citrate (with solid sodium dithionite), and finally, with 0.1 N NaOH for the extraction of Al-P, Fe-P, O-P, and Ca-P.

### 4.3. Statistical Analysis

Data were analyzed using one-way analysis of variance (ANOVA) in SPSS (version 22.0) to determine the significant differences of P on aboveground (maize yield, P uptake, and dry matter biomass) and belowground (RL, SAR, RD, and RA) plant parts, as well as fertilizer use efficiency (REp, FPp, AEp, PHI) among the different treatment groups. The treatments were further subjected to the Duncan Multiple Range Test (DMRT) at a significance level of 95% (*p* < 0.05). The soil S content and forms were illustrated using a dynamic radial bar chart. Microsoft Excel was used to create the graphical tables and figures. A structural equation model (SEM) was constructed using the R multivariate analysis method to explore the direct and indirect effects of aboveground dry matter biomass, P uptake, and root growth indicators on maize yield. All analyses were performed in R (4.1.2).

### 4.4. Formula for Calculating Relevant Indicators

The concentration of P was obtained with the following formulae:

(i) $P=p(\mathrm{ppm})\times \frac{V\times (\frac{V1}{V2})}{wt}$
  where P is extractable, p (ppm) is a concentration from calibration curve, V is the total volume of soil extract (mL), Wt is the weight of dry plant (g), V1 is the volume of soil extract used for measurement (mL), and V2 is the volume of flask used for measurement (mL).
(ii) Root Image Analysis using WinRHIZO was calculated as follows:
  - $RL(m)=\frac{RTW}{RPW}\times TPL$
  - $SAR(m^{2})=\frac{RTW}{RPW}\times TPS$
  - $R\begin{array}{cc} D(mm)= & PAD \end{array}$
  where RL is length of root in treatment (m), SAR is surface area of root in treatment (m^2^), and RD is the diameter of root in treatment (mm);
(iii) REp, % = aboveground P uptake in P application area—aboveground P uptake in non-P application area/P fertilizer application amount;
(iv) FPp, g/g = maize yield/P fertilizer rate;
(v) AEp, g/g = maize yield in P application areas—maize yield in non-P application areas/P fertilizer rate;
(vi) PHI, % = P absorption of maizes/aboveground P absorption × 100%;
(vii) PAC, % = available P/total P × 100%.

## 5. Conclusions

In this study, the different responses of maize plants to various forms of P fertilizers (APP, DAP, FCMP, and DAP+FCMP) were primarily investigated with the goal of assessing the highest P use efficiency in black soils. Our findings demonstrate that APP application significantly increased RA, RL, SAR, and RD, thereby encouraging nutrient uptake and biomass production. These results suggest that different P fertilizers, particularly APP, markedly regulate the morphological and physiological processes of maize roots, enhancing root–soil interactions to increase maize yields. This study highlights the potential to enhance fertilizer utilization efficiency and maize yield in agricultural practices by selecting appropriate fertilizer types eliciting root responses and rhizosphere processes.

## Figures and Tables

**Table 1:** Effects of P fertilizer types on P use efficiency of maize.

Treatment	REp (%)	FPp (g/g)	AEp (g/g)	PHI (%)	PAC (%)
CK	-	-	-	-	6.20 ± 0.16 c
DAP	27.87 ± 1.38 bc	121.14 ± 5.14 b	22.06 ± 5.14 b	48.39 ± 0.17 b	7.45 ± 0.46 b
APP	35.12 ± 3.13 a	132.13 ± 8.63 a	33.05 ± 8.63 a	51.01 ± 1.83 a	8.66 ± 0.11 a
FCMP	29.54 ± 1.04 bc	112.27 ± 2.63 b	13.18 ± 2.63 b	49.06 ± 0.61 b	7.51 ± 0.30 b
DAP+FCMP	24.37 ± 0.20 c	112.63 ± 5.12 b	13.54 ± 5.12 b	48.39 ± 0.32 b	7.43 ± 0.67 b

DAP (diammonium phosphate), APP (ammonium polyphosphate), FCMP (fused calcium magnesium phosphate), DAP+FCMP (diammonium phosphate combined with fused calcium magnesium phosphate), and CK (no phosphate). Different letters indicate significant differences (*p* < 0.05).

## Data Availability

Data are contained within the article.

## Supplementary Materials

The following supporting information can be downloaded at https://www.mdpi.com/article/10.3390/plants13233407/s1, Table S1. Effect of phosphorus fertilizer on maize field yield and phosphorus fertilizer utilization in 2019. Table S2. Pearson correlation is the correlation between biomass, roots, and crop phosphorus uptake, which is derived from the use of different forms of soluble phosphorus fertilizers 120 days after sowing.
