# Supplementary material for: Ammonium Polyphosphate Promotes Maize Growth and Phosphorus Uptake by Altering Root Properties
Source: Plants (Basel). 2024 Dec 4;13(23):3407. doi: 10.3390/plants13233407 (PMC11644354; doi:10.3390/plants13233407)
Supplement: Supplementary file 1 [file plants-13-03407-s001.zip › plants-3252175-supplementary.pdf]

Materials and methods

Experimental design

This experiment was conducted in the experimental field of Jilin Agricultural University (43°48′36″N, 125°24′51″E, altitude 228m). The area is characterized by a continental monsoon climate, with an annual average temperature of 2~12℃ and annual precipitation of 522~615mm.

The field experiment began in 2019, with a total of 6 treatments: 1. Control without phosphorus fertilizer (CK); 2. 100% diammonium phosphate (FP); 3. 25% calcium magnesium phosphate + 75% diammonium phosphate (T1); 4. 50% diammonium phosphate + 50% calcium magnesium phosphate (T2); 5. 75% calcium magnesium phosphate + 25% diammonium phosphate (T3); 6. Ammonium polyphosphate: diammonium phosphate = 2:1 (T4). Each treatment was repeated three times, and the experimental plot area was $48m^2$. The nitrogen application rate for each treatment was 210 kg N/hm^2, the phosphorus application rate was 90 kg P_2O_5/hm^2, and the potassium application rate was 90 kg K_2O/hm^2. Nitrogen fertilizer was used as a base fertilizer at a rate of 30%, and the remaining 70% was applied in two stages as top dressing; all phosphorus and potassium fertilizers were used as base fertilizers. The selected corn variety was Liangyu 99, with a planting density of 65,000 plants/hm^2. Sowing was carried out from late April to early May, and harvesting was done in early October, following the field management practices of farmers for corn cultivation.

Table S1. Effect of phosphorus fertilizer on maize field yield and phosphorus fertilizer utilization in 2019

Treatment	Grain Yield ($t\ ha^{-1}$)	Phosphorus use efficiency (%)
CK	8.64±0.41cd	-
FP	10.17±0.350ab	10.61±2.77b
TI	10.68±0.350a	21.43±1.48a
T2	9.43±0.12c	18.00±5.96a
T3	9.71±0.10bc	16.33±4.10ab
T4	10.53±0.29a	22.24±2.77a

Different letters indicate significant differences ($P < 0.05$). Control without phosphorus fertilizer (CK), 100% diammonium phosphate (FP), 25% calcium magnesium phosphate + 75% diammonium phosphate (T1), 50% diammonium phosphate + 50% calcium magnesium phosphate (T2), 75% calcium magnesium phosphate + 25% diammonium phosphate (T3), Ammonium polyphosphate: diammonium phosphate = 2:1 (T4).

Table S2. Pearson correlation is the correlation between biomass, roots, and crop phosphorus uptake, which is derived from the use of different forms of soluble phosphorus fertilizers 120 days after sowing.

	Pearson's correlation coefficients					
	Biomass	RA	RL	RSA	RD	P uptake
Biomass	1	0.925**	0.686**	0.849**	0.682**	0.836**
RA	0.925**	1	0.675**	0.899*	0.607*	0.839**
RL	0.686**	0.675**	1	0.670**	0.493	0.575*
RSA	0.849**	0.899**	0.670**	1	0.540*	0.863**
RD	0.682**	0.607*	0.493	0.540*	1	0.700**
P uptake	0.836**	0.839**	0.575*	0.863**	0.700**	1

In the table, Biomass: total dry matter mass, RA: root activity, RL: root length, RSA: root surface area, RD: root diameter, P uptake: plant phosphorus uptake. The asterisk indicates significant correlation, * p<0.05, * * p<0.01.
